# Supplementary material for: The Systems Biology Research Tool: evolvable open-source software
Source: BMC Syst Biol. 2008 Jun 29;2:55. doi: 10.1186/1752-0509-2-55 (PMC2446383; doi:10.1186/1752-0509-2-55)
Supplement: Additional file 1 — SBRT Archive. An archive of the current version of the Systems Biology Research Tool. [file 1752-0509-2-55-S1.zip › sbrt-1.4.0/doc/developers_guide/api/sbrt/shell/io/TextInputFile.html]

TextInputFile


|  |  |  |
| --- | --- | --- |
| **Overview** \| **Package** \| **Class** \| **Use** \| **Tree** \| **Deprecated** \| **Index** \| **Help** | |  |
| **PREV CLASS**   **NEXT CLASS** | **FRAMES**    **NO FRAMES**     **All Classes** |  |
| SUMMARY: NESTED \| FIELD \| CONSTR \| METHOD | DETAIL: FIELD \| CONSTR \| METHOD |  |


---


## sbrt.shell.io Class TextInputFile<E>

```
java.lang.Object
  sbrt.shell.io.TextInputFile<E>
```

**Type Parameters:**: `E` - the type contained in instances of this interface.

**All Implemented Interfaces:**: java.lang.Iterable<E>, InputFile<E>, SbrtFile

**Direct Known Subclasses:**: RxnNamExprSetInputFile

---

``` public class TextInputFile<E> extends java.lang.Object implements InputFile<E> ```

This class is used to read input files containing text.

**Author:**
:   This class was written and documented by
    Jeremiah Wright while in the Wagner lab.

---

**Constructor Summary**

`TextInputFile(java.lang.String fileName,
FileFormat fileFormat,
Parser<E> fileLineFormat)`
  
          Constructs a new text input file using the provided arguments.

`TextInputFile(java.lang.String fileName,
FileFormat fileFormat,
Parser<E> fileLineFormat,
java.util.Collection<java.lang.Character> commentCharacters)`
  
          Constructs a new text input file using the provided arguments.

`TextInputFile(java.lang.String fileName,
Parser<E> fileLineFormat)`
  
          Constructs a new text input file using the provided arguments.


| **Method Summary** | |
| --- | --- |
| `void` | `close()`             Closes this file. |
| `protected  void` | `finalize()`             Disposes of system resources associated with this file when called by the garbage collector. |
| `static TextInputFile<java.lang.String>` | `getBasic(java.lang.String fileName)`             Returns a basic text input file. |
| `static TextInputFile<java.lang.String>` | `getBasic(java.lang.String fileName, FileFormat fileFormat)`             Returns a basic text input file. |
| `java.lang.String` | `getCanonicalName()`             Returns the canonical name of this file. |
| `UnmodifiableCollection<java.lang.Character>` | `getCommentChars()`             Returns the commment characters used in this file. |
| `FileFormat` | `getFileFormat()`             Returns the format of this file. |
| `Parser<E>` | `getLineFormat()`             Returns the format used to parse lines of this file. |
| `java.lang.String` | `getOriginalName()`             Returns the name of this file. |
| `boolean` | `isClosed()`             Indicates if this file has already been closed. |
| `static boolean` | `isValidFormat(FileFormat fileFormat)`             Indicates if the provided file format is valid for a text input file. |
| `java.util.Iterator<E>` | `iterator()`             Returns a line iterator over this text file. |
| `java.util.Iterator<E>` | `iterator(int line)`             Returns a line iterator over this text file beginning at the specified line. |
| `protected  TextFileLineIterator<E>` | `lineIterator()`             Returns a line iterator over this text file. |

**Methods inherited from class java.lang.Object**

`clone, equals, getClass, hashCode, notify, notifyAll, toString, wait, wait, wait`

**Constructor Detail**

### TextInputFile

```
public TextInputFile(java.lang.String fileName,
                     Parser<E> fileLineFormat)
```

:   Constructs a new text input file using the provided arguments.

    **Parameters:**: `fileName` - the name of the file to be read.: `fileLineFormat` - the format used to parse the lines of this file.

---


### TextInputFile

```
public TextInputFile(java.lang.String fileName,
                     FileFormat fileFormat,
                     Parser<E> fileLineFormat)
```

:   Constructs a new text input file using the provided arguments.

    **Parameters:**: `fileName` - the name of the file to be read.: `fileFormat` - the format of the file to be read.: `fileLineFormat` - the format used to parse the lines of this file.

---


### TextInputFile

```
public TextInputFile(java.lang.String fileName,
                     FileFormat fileFormat,
                     Parser<E> fileLineFormat,
                     java.util.Collection<java.lang.Character> commentCharacters)
```

:   Constructs a new text input file using the provided arguments.

    **Parameters:**: `fileName` - the name of the file to be read.: `fileFormat` - the format of the file to be read.: `fileLineFormat` - the format used to parse the lines of this file.: `commentCharacters` - a collection of characters used for comments in this file.


**Method Detail**

### getBasic

```
public static TextInputFile<java.lang.String> getBasic(java.lang.String fileName)
```

:   Returns a basic text input file.

    :   **Parameters:**: `fileName` - the name of the file to be read. **Returns:**: a basic text input file.

---


### getBasic

```
public static TextInputFile<java.lang.String> getBasic(java.lang.String fileName,
                                                       FileFormat fileFormat)
```

:   Returns a basic text input file.

    :   **Parameters:**: `fileName` - the name of the file to be read.: `fileFormat` - the file format of this file. **Returns:**: a basic text input file.

---


### isValidFormat

```
public static boolean isValidFormat(FileFormat fileFormat)
```

:   Indicates if the provided file format is valid for
    a text input file.

    :   **Parameters:**: `fileFormat` - the file format to test. **Returns:**: `true` if the provided file format is valid for a text input file; `false` otherwise.

---


### getLineFormat

```
public Parser<E> getLineFormat()
```

:   Returns the format used to parse lines of this file.

    :   **Returns:**: the format used to parse lines of this file.

---


### getFileFormat

```
public FileFormat getFileFormat()
```

:   Returns the format of this file.

    :   **Returns:**: the format of this file.

---


### getCommentChars

```
public final UnmodifiableCollection<java.lang.Character> getCommentChars()
```

:   Returns the commment characters used in this file.

    :   **Returns:**: the commment characters used in this file.

---


### getOriginalName

```
public final java.lang.String getOriginalName()
```

:   Returns the name of this file.

    :   **Specified by:**: `getOriginalName` in interface `SbrtFile`
    :   **Returns:**: the name of this file.

---


### getCanonicalName

```
public final java.lang.String getCanonicalName()
```

:   Returns the canonical name of this file.

    :   **Specified by:**: `getCanonicalName` in interface `SbrtFile`
    :   **Returns:**: the canonical name of this file. **See Also:**: `File.getCanonicalPath()`

---


### lineIterator

```
protected final TextFileLineIterator<E> lineIterator()
```

:   Returns a line iterator over this text file.

    :   **Returns:**: a line iterator over this text file.

---


### iterator

```
public java.util.Iterator<E> iterator()
```

:   Returns a line iterator over this text file.

    :   **Specified by:**: `iterator` in interface `java.lang.Iterable<E>`
    :   **Returns:**: a line iterator over this text file.

---


### iterator

```
public java.util.Iterator<E> iterator(int line)
```

:   Returns a line iterator over this text file beginning at the specified
    line. Line indices begin at `0`, and commented or blank lines are
    ignored.

    :   **Parameters:**: `line` - the index of the line at which this iterator begins. **Returns:**: a line iterator over this text file beginning at the specified line. **Throws:**: `java.lang.IndexOutOfBoundsException` - if the provided number exceeds the number of lines contained in the file.

---


### close

```
public final void close()
```

:   Closes this file. If this file is already closed, then
    invoking this method has no effect.

    :   **Specified by:**: `close` in interface `SbrtFile`
    :   **Throws:**: `ApplicationException` - if an I/O error occurred while closing this file.

---


### isClosed

```
public final boolean isClosed()
```

:   Indicates if this file has already been closed.

    :   **Specified by:**: `isClosed` in interface `SbrtFile`
    :   **Returns:**: `true` if this file is closed; `false` othewise.

---


### finalize

```
protected final void finalize()
                       throws java.lang.Throwable
```

:   Disposes of system resources associated with this file
    when called by the garbage collector.

    :   **Overrides:**: `finalize` in class `java.lang.Object`
    :   **Throws:**: `java.lang.Throwable` - the Exception raised by this method. **See Also:**: `Object.finalize()`


---


|  |  |  |
| --- | --- | --- |
| **Overview** \| **Package** \| **Class** \| **Use** \| **Tree** \| **Deprecated** \| **Index** \| **Help** | |  |
| **PREV CLASS**   **NEXT CLASS** | **FRAMES**    **NO FRAMES**     **All Classes** |  |
| SUMMARY: NESTED \| FIELD \| CONSTR \| METHOD | DETAIL: FIELD \| CONSTR \| METHOD |  |


---
